# Correlation between the implementation of Psychosocial Care Centers and the rates of psychiatric hospitalizations and suicide in Porto Alegre-RS from 2008 to 2018

**DOI:** 10.47626/2237-6089-2021-0220

**Published:** 2023-05-02

**Authors:** José Milton Alves dos Santos, Karoline Kuczynski, Caroline Vicenzi, Alexandre Lorini, Karen Jansen, Coral Rakovski

**Affiliations:** 1 Universidade Católica de Pelotas Pelotas RS Brazil Universidade Católica de Pelotas (UCPel), Pelotas, RS, Brazil.; 2 Universidade Federal de Pelotas Pelotas RS Brazil Universidade Federal de Pelotas (UFPel), Pelotas, RS, Brazil.; 3 Neuroscience Graduate Program McMaster University Hamilton ON Canada Neuroscience Graduate Program, McMaster University, Hamilton, ON, Canada.

**Keywords:** Mental health, community mental health centers, hospitalization, suicide

## Abstract

**Introduction:**

The Brazilian psychiatric reform has revolutionized the way that mental health care is provided all over the country, introducing the Psychosocial Care Centers (CAPS) and encouraging care at liberty. The CAPS have been assigned many objectives, such as prevention of hospitalizations and intervention in crises or suicide. This paper aims to describe the correlation between the implementation of CAPS and the rates of psychiatric hospitalizations and suicides from 2008 to 2018.

**Methods:**

This study has an ecological time series design and included residents of the city of Porto Alegre, RS, Brazil, who were hospitalized through the Sistema Único de Saúde (SUS). The data were obtained from official databases (DATASUS, CNES, and IBGE) and indicators were calculated (CAPS coverage, hospitalization rate, and suicide rate). Associations between the indicators were tested using Pearson’s correlation coefficients.

**Results:**

We found a negative correlation between provision of CAPS and psychiatric hospitalizations (r = -0.607 p = 0.048).

**Conclusion:**

These results support the hypothesis that there is a negative correlation between implementation of the CAPS and psychiatric hospitalizations. This reinforces the importance of implementing policies related to improving psychiatric reform.

## Introduction

Taking 1970 as a starting point, the Brazilian psychiatric reform has its roots in social movements and young graduates who questioned the mental health care provided up to that point, mainly with regards to hospitalization in psychiatric hospitals. It was argued that deinstitutionalization of patients could be achieved by providing services to substitute prolonged hospitalizations.^1^ A major milestone in the reform process was the passing of Law No. 10,216, in 2001,^2^ which constituted the first step in change to the mental health care model. Subsequently, the Psychosocial Care Centers (CAPS) were created as the central service in provision of this care, now with patients at liberty. Ordinance No. 336, of 2002,^3^ designates the CAPS as territorial and community-based outpatient services. On December 23, 2011, Ordinance 3.088 was passed, establishing a mental health care network, the Psychosocial Care Network (RAPS), thus creating, expanding, and articulating mental health care within the Unified Health System (SUS).^4,5^

The CAPS are primarily responsible for treating individuals presenting with psychological distress/mental disorders, including those resulting from use of alcohol and other drugs. The CAPS can be of various types, according to their objectives: from CAPS I, which serves regions with populations of over 15 thousand, to child CAPS, which are for providing care to children and adolescents in regions with populations of over 70 thousand inhabitants. Some of the most important objectives of the CAPS are providing primary care for serious and persistent disorders and promoting the social inclusion and autonomy of users.^6,7^ It is envisaged that the CAPS will employ preventive measures, so that hospital admissions are only required in specific cases. Implementation of preventative measures is crucial for addressing crises, including suicidal ideation, and for provision of adequate care in such situations.^8^

Hospitalization is resorted to when alternative outpatient hospital interventions are insufficient for treatment, such as at times when participants are experiencing severe mental health challenges and are at risk of harming themselves or others. Hospitalizations are typically of short duration (1-2 weeks), especially when admission is to general hospitals, and hospitalization is used in conjunction with other RAPS services.^9,10^ Suicide risk should also be managed in the CAPS, preventing escalation to suicide. Approximately 28% of CAPS users are at high risk of committing suicide, demonstrating the importance of these services for preventing adverse outcomes, such as death.^11,12^

Nineteen years after implementation of the CAPS, there are still frequent discussions regarding the importance of these services and also with regard to the Brazilian psychiatric reform in general. However, few studies have investigated the impact of these services. Thus, this article aims to assess the correlation between implementation of the CAPS and the rates of psychiatric hospitalizations and death by suicide in Porto Alegre, RS, Brazil, from 2008 to 2018. It was hypothesized that there would be a statistically significant, negative correlation between the number of CAPS and the rates of psychiatric hospitalizations and suicide.

## Methods

This study has an ecological time series design, which is appropriate for observation of the effects resulting from implementation of the CAPS. The period from 2008 to 2018 was delimited by the availability of data. The study population comprised residents of the city of Porto Alegre, the capital of Rio Grande do Sul, who were admitted by the SUS.

Data were obtained through the SUS Hospital Information System (SIH-SUS), the National Registry of Health Facilities (CNES), and the Demographic Census. These data are available on the DATASUS (https://datasus.saude.gov.br/) and from the Brazilian Institute of Geography and Statistics (IBGE) (https://www.ibge.gov.br/) websites. Information was collected regarding the number of CAPS, the number of psychiatric hospitalizations coded as ICD 10 codes F20 to F39, and the resident population of Porto Alegre.

The main diagnosis assigned to each hospitalization was used, according to International Statistical Classification of Diseases and Related Health Problems - 10th Edition (ICD-10) codes, selecting clusters F20 to F29 (schizophrenia, schizotypical, and delusional disorders) and F30 to F39 (mood/affective disorders). For convenience, and due to the aim of observing a specific group of illnesses, codes F10 to F19 (mental and behavioural disorders due to the use of psychoactive substances) were excluded. For the suicide data, X.70 and X.80 (intentional self-harm) were grouped together.

Porto Alegre was selected for this study because the data are of a higher quality than that data available for other cities. As recommended by the Ministry of Health, the “CAPS coverage rate per 100 thousand inhabitants” (or CAPS coefficient) was used as the parameter for CAPS coverage in RAPS.^9^ The psychiatric hospitalization rate was calculated using the ratio between the absolute number of hospitalizations and the resident population multiplied by 100 thousand inhabitants. The suicide rate was also calculated using the ratio between the number of suicides and the resident population multiplied by 100 thousand inhabitants.

The data were obtained entirely from official databases available on the internet, thus minimizing risks for individuals and ensuring that the study was conducted in accordance with ethical standards.

Microsoft Excel version 16.0 and IBM SPSS version 16.0 were used to organize data and for statistical analysis. Variables were expressed as relative and absolute frequencies or means and standard deviations. Since the variables were numerical, Pearson’s correlation coefficients were used to test the hypotheses. The significance level was set at 5%.

## Results

In 10 years (2008-2018) the population of Porto Alegre grew by 1.4% (Table 1). Over the same period, there was a 24% reduction in psychiatric hospitalizations, where psychotic and mood disorders accounted for 28% and 21% of the total reduction, respectively.

**Table 1:** Population, hospitalization rates, suicide rates, and CAPS coefficients in Porto Alegre (2008 to 2018)

Year/ information	Population	Psychiatric hospitalizations	Psychotic disorders			Mood disorders			Suicide			CAPS coefficient
			Total	Male	Female	Total	Male	Female	Total	Male	Female	
2008	1444697	227	84	56	28	143	46	97	6.78	5.05	1.73	0.2
2009	1452417	244	94	65	29	150	52	98	6.46	5.71	0.75	0.2
2010	1437588	260	97	69	28	163	56	107	6.94	5.35	1.59	0.2
2011	1445787	264	99	71	28	165	58	106	5.79	4.14	1.65	0.3
2012	1454435	262	105	71	34	157	54	103	5.29	4.19	1.1	0.5
2013	1464037	254	105	64	41	149	45	105	7.37	5.46	1.91	0.5
2014	1467525	221	90	58	32	131	39	92	7.48	5.51	1.97	0.6
2015	1465428	190	73	45	29	116	32	85	6.68	5.18	1.5	0.6
2016	1464231	192	72	45	26	120	32	88	6.54	4.91	1.63	0.6
2017	1468301	186	66	41	25	120	30	90	7.42	6.06	1.36	0.6
2018	1465430	172	60	37	23	112	29	83	6.54	4.57	1.97	0.6

CAPS = Psychosocial Care Centers.

In 2008, the CAPS coverage coefficient was 0.2, while by 2018 it had increased to 0.6, representing an increase of 300%. Analysis of the correlation between the CAPS coverage ratio and the rate of psychiatric hospitalizations showed a negative and statistically significant coefficient (r = 0.607; p = 0.048). There was no significant correlation between the rate of hospitalizations for psychotic disorders and the coverage ratio of CAPS. However, there was a significant correlation between the CAPS coefficient and the rate of hospitalizations for mood disorders (r = -0.698; p = 0.017) (Table 2).

**Table 2:** Correlations between CAPS coefficient, psychiatric hospitalization rates, and suicide rates in Porto Alegre (2008 to 2018)

	R coefficient	p value
Psychiatric	-0.607	0.048*
Total psychotic	-0.462	0.152
Male psychotic	-0.616	0.044*
Female psychotic	0.083	0.809
Total mood	-0.698	0.017*
Male mood	-0.757	0.007*
Female mood	-0.590	0.560
Total suicide	0.343	0.302
Male suicide	0.115	0.737
Female suicide	0.194	0.568

CAPS = Psychosocial Care Centers.

* Statistical significance at the level of 5%.

Coefficients were negative for the correlations between the CAPS coverage coefficient and the hospitalization rate for men with psychotic disorders (r = -0.616; p = 0.044) and men with mood disorders (r = -0.757; p = 0.007). There was no correlation between the CAPS coverage coefficient and the rate of hospitalization of women with psychotic and mood disorders (Table 2).

**Figure 1:**
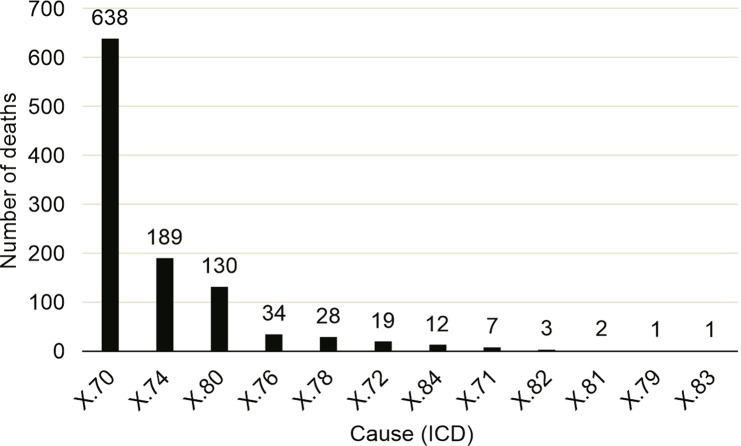
Number of deaths by cause (according to ICD-10) in Porto Alegre from 2008 to 2018.

**Figure 2:**
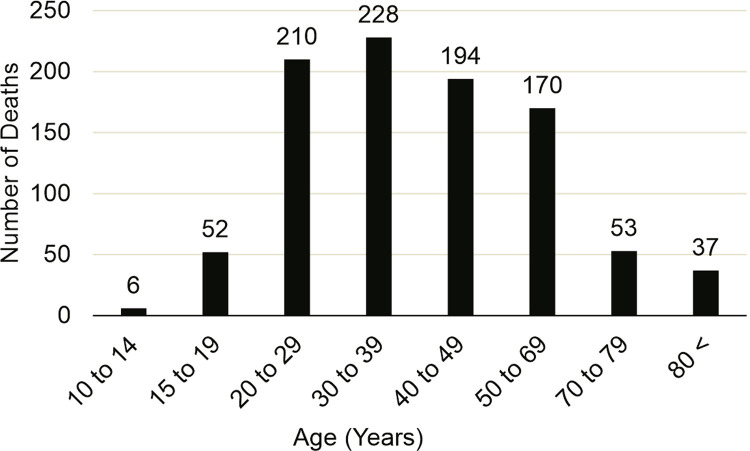
Number of deaths by age group in Porto Alegre from 2008 to 2018.

During the same period, we found a total of 1,070 suicide deaths, approximately 76% of which were deaths of males. Regarding age groups, there were 228 deaths (21%) in the 30-39 years age group. The most common type of suicide was that of Intentionally Self-Injured Injury (LAI) due to hanging, strangulation, and suffocation (X.70), with 638 deaths (59%). The year with the highest prevalence of deaths by suicide was 2014, accounting for 10% of the cases (Figure 1). It was noted that the suicide rate varied slightly over the 10 years (2008-18), but remained close to six deaths per 100 thousand inhabitants (6.64±0.674). The average number of suicide deaths over the 10 years was 5.1 (±0.701) for men and 1.5 (±0.467) for women (Figure 2).

There was no statistically significant correlation between the CAPS coefficient and the total suicide rates, for either men or women.

## Discussion

Implementation of the CAPS in Porto Alegre, RS, between 2008 and 2018, reduced psychiatric hospitalizations. It also decreased hospitalizations for patients with mood disorders. Regarding the subgroups, these services mitigated hospitalizations for men with psychotic and mood disorders.

In recent years, population growth in Brazil has decreased, although it still remains high,^13^ contrasting with the rate in Porto Alegre, which has been growing more slowly (2.4%). Despite this, the number of psychiatric hospitalizations decreased by 24%, highlighting the reduction in hospitalizations for psychotic disorders. This is similar to the results of another study,^14^ which found a 7% reduction in hospitalization rates among this group from 2001 to 2013.

After analyzing the implementation of CAPS in this period and its coefficient or rate of assistance coverage, we observed a large increase, from 0.2 in 2008 to 0.6 in 2018. According to the Ministry of Health,^8^ we can define the rate of care coverage per 100 thousand inhabitants as very good (above 0.70), good (0.50 to 0.69), regular (between 0.35 and 0.49), low (between 0.19 and 0.34), or insufficient/critical (below 0.20).^15^ The city of Porto Alegre is no longer within the critical classification, which is a result that has also been reported by Costa et al.^5^ Furthermore, the great increase in implementation of these services is in line with the mental health policy of the period,^16^ as observed in several Brazilian cities such as Rio de Janeiro, where another study pointed to a significant increase in implementation of CAPS in the years prior to 2015.^17^

The CAPS are mainly an alternative to psychiatric hospitalization and they are charged with working to further the social reintegration and autonomy of their users. The CAPS also use preventative measures to avoid hospitalizations whenever possible.^18^ This study found that an increase in CAPS coverage was associated with a reduction in the rate of psychiatric hospitalizations. A similar trend was found in the cities of São Paulo and Rio de Janeiro, which also observed a reduction in psychiatric hospitalizations after an increase in the number of Psychosocial Care Centers.^19^

Upon stratifying the analysis, we found a correlation between CAPS coverage rates and the rates of hospitalizations for mood disorders, but this was not observed for hospitalizations for psychotic disorders. De Araújo et al.^20^ reports the recurrence of hospitalizations and the difficulties that patients in this second group have in adhering to the service. In addition, another author also observed low drug adherence in psychotic patients receiving the service.^21^ On the other hand, Fonteles et al.^22^ observed good adherence to treatment in patients with mood disorders.

We also observed differences in the correlation between CAPS coverage and admission rates by sex. In males, we observed that implementation of the CAPS reduced hospitalizations both for psychotic disorders and for mood disorders, but this situation was not observed in females. Although studies differ with regard to the sex proportion of the users of these services, overall they point to greater adherence to treatment by men than by women,^23,24^ highlighting situations that make it difficult for women to adhere, such as excessive working hours and even greater severity in some pathologies.^25^ Another hypothesis is that the majority of women receiving treatment from CAPS present with a moderate or mild condition, which rarely requires psychiatric hospitalization.^21^

Porto Alegre showed a relatively constant suicide rate in our study, predominantly among the 30-to-39 years age group. This trend is different from that observed in the state of Rio Grande do Sul by Franck et al.,^26^ who found an increase in suicide rates and a higher prevalence in the 50-54 years age range in males and the 50-59 years age range in females. The differences found in suicide rates between the sexes (higher in men) are also in line with other studies. The Ministry of Health^27^ reports a suicide rate that is 4 times higher in men than in women. It also points to a relatively stable suicide rate in recent years.

There was no statistically significant correlation between implementation of the CAPS and the reduction in suicide rates. These results differ from those of another study, which showed a 57% reduction in deaths by suicide after an increase in the CAPS coverage rate.^28^ It is possible that this result is a reflection of difficulties encountered by services, such as overcrowding, insufficient number of adequate workers, and location.^29,30^ Alternatively, this observation could be subject to bias, because the majority of users who commit suicide are not served by the CAPS, and this condition (suicide) is not directly associated with these centers.^31^

In addition, when analyzing suicide rates, it should be noted that other RAPS services (such as primary health care and hospital care) also serve individuals at risk of suicide and prevent hospitalizations.^32^ Suicide risk is also influenced by social and economic issues, including risk factors such as living alone or experiencing economic difficulties.^33^

One limiting factor of this study was that it did not include an analysis of the number of psychiatric beds and the corresponding trends observed during the study period. According to the Federal Council of Medicine,^34^ the state of Rio Grande do Sul had a 140% increase in the number of psychiatric beds from 2005 to 2016, with 2,184 beds at the end of that period. This trend is different from that observed for the country as a whole, which had a reduction in the number of beds. Thus, we have demonstrated that even with an increase in beds, hospitalization rates for some groups reduced. Notwithstanding, we note that, based on the results of this study, implementation of the CAPS shows significant promise for care of psychiatric patients in Porto Alegre.

## Conclusion

These findings support the hypothesis of a negative correlation between implementation of the CAPS and a decrease in psychiatric hospitalization rates. This was observed in the number of hospitalizations for mood disorders (ICD10 F30-F39) and among men (hospitalized for ICD10 F20 to F39), from 2008 to 2018 in Porto Alegre. These results reaffirm the need for continuity in and improvement of policies associated with psychiatric reform. Other studies, incorporating different designs, may further elucidate important information regarding the Brazilian psychiatric reform.
